# Dihydrotestosterone Promotes Hetian Sheep Dermal Papilla Cell Proliferation by Inhibiting the TGF‐β/Smad Signalling Pathway

**DOI:** 10.1002/vms3.71118

**Published:** 2026-09-23

**Authors:** Qinqin Wang, Xuan Liu, Zhiya Feng, Jianping Zhang, Chunyang Li, Ruijun Shi, Shuwei Li

**Affiliations:** ^1^ State Key Laboratory Incubation Base for Conservation and Utilization of Bio‐Resource in Tarim Basin, College of Life Science and Technology Tarim University Alar China

**Keywords:** cell proliferation, dermal papilla cell, dihydrotestosterone, Hetian sheep, TGF‐β/Smad signalling pathway

## Abstract

Hair follicles, as key accessory organs in mammalian skin, are precisely regulated for growth and development influenced by genetic factors, hormones, and the local microenvironment. This study investigated the effect of dihydrotestosterone (DHT) on the proliferation of dermal papilla cells (DPCs) from Xinjiang Hetian sheep and its regulatory relationship with the TGF‐β/Smad signalling pathway. DPCs were isolated from the back skin tissue of newborn Hetian sheep by collagenase II digestion, and their characteristics were identified by primary culture, passage, and double immunofluorescence staining with *α*‐SMA and CD133 labelling. CCK‐8 and EdU assays were used to evaluate the effect of varying DHT concentrations (0, 1, 10, 100, and 1000 nM) on DPC proliferation. The results showed that with the increased DHT concentration, cell proliferation also increased, and the highest rate was observed at 1000 nM. Furthermore, qPCR analysis revealed that 1000 nM DHT significantly upregulated *Smad6* gene expression (*p* < 0.05), while it inhibited the expression of *TGF‐β1, TGF‐βR*II, and *Smad5* (*p* < 0.05). These findings suggest that high DHT concentrations may promote DPC proliferation by inhibiting the TGF‐β/Smad signalling pathway. This suggests a critical role of this pathway in regulating hair follicle development and hormone‐dependent hair growth, providing evidence for the molecular mechanism of androgen action on dermal papilla cell function.

## Graphical abstract

This study successfully isolated Dermal Papilla Cells (DPCs) from Hetian sheep and established a stable in vitro culture system for these cells. It was found that androgens promote the proliferation of Hetian sheep DPCs by inhibiting the expression of genes in the TGF‐β/Smad signalling pathway, thereby influencing hair follicle development. Furthermore, an in vitro model for sheep‐based research was established, which bridges androgen regulation levels with hair follicle development

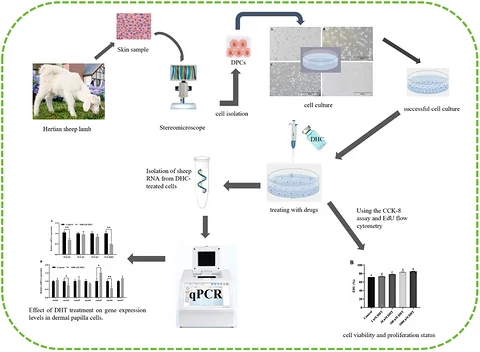

## 1 Introduction

Hair follicles are key accessory organs in mammalian skin, and their growth and development are influenced by genetic factors, hormones, and the local microenvironment. A hair follicle consists of several structures, including the inner root sheath, outer root sheath, connective tissue sheath, hair bulb, and dermal papilla. The dermal papilla, located at the base of the hair bulb, is rich in blood vessels and nerves, which supply nutrients to support hair growth. The inner and outer root sheaths protect the hair shaft and participate in regulating the hair growth cycle. The connective tissue sheath serves to fix and protect the hair follicle (Zhang et al. 2022; Zhang et al. 2024; Oppenheimer et al. 2024). Dermal papilla cells (DPCs), located within the hair bulb, exhibit specific heterogeneity and characteristics similar to adult stem cells (Hirose et al. 2025). As central regulators of signals governing hair follicle development and cyclic growth, DPCs release various signalling molecules through paracrine mechanisms. These molecules guide the proliferation and upward migration of surrounding follicular matrix cells. The migrating cells then differentiate into the hair bulb, inner root sheath, and outer root sheath structures (Kageyama et al. 2023; Burnat et al. 2025; Lee et al. 2024). Consequently, DPCs modulate cyclic changes in hair follicle growth and thereby maintain and promote healthy follicle development. DPCs also interact with epithelial cells by secreting multiple growth factors (such as VEGF, FGF, and Wnt). These factors sustain the activity of hair follicle stem cells and drive hair growth (Ying et al. 2024). Research indicates that dysfunction in DPCs is closely associated with pathological processes such as alopecia and follicle atrophy. Understanding the regulatory mechanisms of DPC proliferation is therefore crucial for studying hair follicle development and designing therapeutic strategies for hair regeneration.

Hair growth and follicle development are strongly influenced by hormones. Androgens play a critical role in hair growth regulation. Dihydrotestosterone (DHT), a potent androgen, exhibits a dual effect on hair growth. While DHT promotes the growth of body hair (such as beards and underarm hair) (Burnat et al. 2025), its excessive accumulation leads to androgenetic alopecia. This condition is closely linked to the influence of DHT on DPCs in the scalp. Current studies suggest that DHT may alter the balance between proliferation and apoptosis of DPCs by modulating signalling pathways such as Wnt/β‐catenin and TGF‐β/Smad. However, the precise mechanisms remain incompletely understood.

Transforming growth factor‐β (TGF‐β), a multifunctional growth factor, is a profibrotic cytokine (Darjani et al. 2023; Ayça et al. 2015; Yao et al. ). It can induce fibroblast proliferation and transformation, playing a key role in fibrosis formation. TGF‐β exerts diverse effects across a broad spectrum of cellular processes. The TGF‐β superfamily includes over 30 ligands that belong to subfamilies such as TGF‐β, bone morphogenetic proteins, activins, inhibins, growth differentiation factors, and nodals. In the canonical TGF‐β signalling pathway, TGF‐β ligands bind specific serine/threonine kinase receptors (type I and type II). These receptors trigger the activation of receptor‐specific Smads (Aftabi et al. 2025). Activated Smads form heteromeric complexes with a common mediator Smad, named Smad4. Smad proteins are classified into three categories: receptor‐regulated Smads (e.g., *Smad2* and *Smad3*), common Smads (e.g., *Smad4*), and inhibitory Smads (e.g., *Smad7*) (Darjani et al. 2023; Ayça et al. 2015; Yao et al. ). Studies show that the TGF‐β signalling pathway regulates epidermal homeostasis, hair cycle progression, and the proliferation and differentiation of epithelial stem cell populations in hair follicles. The TGF‐β/Smad transduction system involves membrane receptor kinases and Smad protein substrates (Ong et al. 2021). After binding to its receptor complex, TGF‐β activates Smad proteins. These activated Smads form multi‐subunit complexes, enter the nucleus, and regulate gene transcription (Ong et al. 2021; Yao et al. 2021; Peng et al. 2022). During TGF‐β stimulation, the combination of Smad partners and regulatory factors determines the specific cellular response. Among these, *Smad1* and *Smad5* are unique TGF‐β pathway mediators that regulate hair follicle development, cyclic growth, and pigment deposition. It is known that androgens promote TGF‐β1 expression in human DPCs. Therefore, other isoforms and receptors of TGF‐β in DPCs may also be androgen‐sensitive (Yao et al. 2021; Umbarkar et al. 2019; Lee et al. 2024). Androgens may inhibit TGF‐β signalling in sheep DPCs, thereby affecting hair follicle morphogenesis. However, whether androgens regulate gene expression in the TGF‐β*/*Smad signalling pathway requires further investigation.

The Hetian sheep, an indigenous breed from the Hetian area of Xinjiang, China, is well recognised for its high‐quality wool and strong adaptability to extreme environments, making it a valuable biological resource. In contrast to conventional animal cell models such as rabbits and mice, primary cells derived from neonatal Hetian lambs show significantly higher viability and improved proliferative capacity. Neonatal lambs were selected as the optimal donor model for several key reasons: their dermal papilla cells (DPCs) exhibit superior viability and proliferative potential, which are crucial for establishing robust primary cultures; their low endogenous androgen levels minimise confounding hormonal interference, thereby allowing a more precise evaluation of the direct effects of exogenous dihydrotestosterone (DHT); and the use of a uniform neonatal age group ensures greater biological consistency and experimental reproducibility across all samples. These cells serve as a more reliable model system for investigating the mechanisms that drive the development of premium wool fibre characteristics, thereby directly supporting the Hetian sheep industry and increasing the economic value of wool products. Furthermore, as the first established culture of Hetian sheep cells, this system provides unique and irreplaceable advantages for both basic and applied biological research. Notably, ovine skin is similar to human skin in terms of structure, thickness, follicular density, and cyclic growth patterns, especially in processes such as wound healing and scar formation. This similarity highlights the relevance of the Hetian sheep cell model as a tool for biomedical studies on human dermatological and trichological disorders (Umbarkar et al. 2019). This study uses Hetian sheep as a model to investigate the effects of DHT on DPC proliferation and its relationship with the TGF‐β/Smad pathway. The goal is to clarify the role of the TGF‐β/Smad pathway in hair follicle development and hormone‐dependent hair growth (Elkiki et al. 2021). We isolated and cultured DPCs from Hetian sheep and applied cell and molecular biology techniques to assess the dose‐dependent effects of DHT on cell proliferation. We also analysed changes in gene expression related to TGF‐β family members and Smad signalling.

Studies have demonstrated that CD133 serves as a surface‐specific marker of DPCs, exhibiting high expression levels in human and murine DPCs, while being minimally expressed or absent in other skin cell types (e.g., fibroblasts and epidermal cells). In hair follicle reconstitution assays, CD133‐high DPCs show enhanced efficacy in inducing epithelial cells to form new follicular structures, establishing CD133 as a critical indicator for evaluating DPC functional activity (Peng et al. 2022; Umbarkar et al. 2019; Lee et al. 2024). *α*‐smooth muscle actin (*α*‐SMA) is a classical marker of myofibroblasts and vascular smooth muscle cells. Its expression in DPCs is associated with contractile properties and functions related to maintaining follicular structure. In hair loss disorders such as androgenetic alopecia, aberrant increased expression of *α*‐SMA in DPCs may contribute to perifollicular fibrosis or functional degeneration, thereby providing important insights into pathological mechanisms (Umbarkar et al. 2019; Elkiki et al. 2021).

This study employs Hetian sheep as a model to investigate the regulatory mechanism by which dihydrotestosterone (DHT) modulates dermal papilla cell proliferation via the TGF‐β/Smad signalling pathway. The findings provide direct evidence for elucidating the biological basis of wool fibre quality formation and also offer novel insights and potential therapeutic targets for research on human hair disorders, such as androgenetic alopecia. The research outcomes hold significant theoretical and practical implications for promoting the high‐value utilisation of characteristic livestock genetic resources and advancing human hair regenerative medicine.

## 2 Materials and Methods

### 2.1 Animals

The samples used in this study were 3‐day‐old female lambs provided by the Institutional Animal Experiment Center. All experimental protocols were approved by the relevant institutional committee (approval number: PB20260112003). The dorsal wool of the lambs was shaved to expose a 15 × 15 cm area of skin, which was disinfected with iodine tincture and 75% ethanol. A subcutaneous infiltration of 2% lidocaine (3–4 mg/kg) was administered. Under strict aseptic conditions, a full‐thickness skin sample measuring 2 × 2 cm was excised using sterile scissors. The excised tissue was rinsed gently three times with PBS (Phosphate Buffered Saline containing) 100 U/mL penicillin and 0.1 mg/mL streptomycin, and then placed in Dulbecco's Modified Eagle Medium/Nutrient Mixture F‐12 (DMEM/F‐12, Gibco, D6570) medium and stored at 4°C. Haemostasis was achieved by applying gentle pressure with sterile gauze for 3–5 min. The wound was irrigated with 0.9% saline, disinfected with povidone‐iodine, and evenly sprayed with a topical antibiotic ointment containing gentamicin. A sterile dressing was applied and secured with an elastic bandage. The dressing integrity and the wound condition, including redness, swelling, exudation, or odour, were monitored daily. The lambs were housed individually in pens with dry and clean soft bedding to prevent friction. The dressings were removed after 10 days, and the wounds were observed until complete healing.

### 2.2 Isolation and Culture of DPCs

DPCs were isolated from the dorsal skin of one healthy Hetian female lamb. To minimise operational error and ensure the reliability of results, three independent tissue blocks were obtained from this donor for primary cell isolation. The skin specimens were placed in a biosafety cabinet, and the external packaging was disinfected with 75% ethanol before transfer to the sterile working area. The tissues were rinsed three times (2 min each) with ice‐cold PBS containing 1% penicillin–streptomycin. Using sterile microdissection forceps, adipose and connective tissues were carefully removed in DMEM/F‐12 medium pre‐chilled on ice. The remaining dermal tissue was minced into 2–4 mm fragments. The fragments were transferred into a 15 mL centrifuge tube, and 8 mL of serum‐free DMEM/F‐12 medium (pH 7.4) containing 0.25 mg/mL collagenase type II (Solarbio, C8150‐100) was added, resulting in a tissue‐to‐enzyme solution ratio of 1:8. The mixture was digested for 2 h at 37°C with continuous shaking at 120 rpm. The tube was gently agitated every 30 min to prevent localised overheating.

Following digestion, the enzymatic reaction was terminated by adding DMEM/F‐12 supplemented with 10% fetal bovine serum (FBS, Gibco, S9020). The mixture was gently triturated 10 times using a 1 mL wide‐bore pipette tip to dissociate hair follicles from dermal tissue. The resulting suspension was filtered through a 70 µm cell strainer into a new tube and centrifuged at 1500 rpm for 5 min at 4°C. The supernatant was carefully discarded, and the cell pellet containing intact hair follicles was obtained.

Microdissection and primary culture of DPCs: The pellet containing intact hair follicles was resuspended in ice‐cold DMEM/F‐12 medium and transferred dropwise onto the stage of a dissection microscope (Motic, SMZ‐171), which was placed on an ice pack to maintain a low temperature. Under 10× to 40× magnification, the hair bulb was stabilised with No. 5 microforceps. The narrowest region of the hair bulb (the dermal papilla neck) was circumferentially incised using micro‐spring scissors (blade length: 5 mm), preserving only the natural connection between the bulb and dermal papilla. A glass capillary tube with an inner diameter of 0.2 mm was then used to aspirate a microdroplet of DMEM/F‐12 medium. The droplet was gently brought into contact with the dermal papilla, facilitating its complete detachment via surface tension. Additional medium was added as needed to prevent desiccation. Each isolated dermal papilla was immediately transferred to an individual well of a 96‐well plate containing complete medium (DMEM/F‐12 supplemented with 10% FBS and 1% penicillin–streptomycin) to avoid drying and mechanical stress. Cells from the same tissue block were cultured in three technical replicate wells, resulting in a total of nine wells from a single biological individual. Following this plating protocol, the culture plate was placed in a 37°C, 5% CO_2_ incubator for primary culture. The cultures were monitored daily under an inverted microscope for tissue attachment and cell migration. After 5 days of culture, polygonal cells were observed migrating from the explants, forming a growth halo that covered approximately 50% of the well surface area. Following this initial culture period, the medium from all wells containing migrating DPCs was pooled into a 15 mL conical tube and centrifuged at 1500 rpm for 5 min. The supernatant was discarded, and the cell pellet was resuspended in fresh complete medium and seeded into a 60 mm culture dish for subsequent expansion. All procedures were carried out under low‐temperature conditions with strict avoidance of desiccation. When the cells reached approximately 80% confluency, they were passaged. All nine wells of cells were successfully cultured in vitro.

Therefore, all in vitro experiments in this study were derived from DPCs of a single biological donor (*n* = 1). Throughout the manuscript, the term ‘replicates’ refers exclusively to technical replicates (e.g., multiple wells, dishes, or qPCR reactions from the same cell pool), which were employed to assess measurement precision and experimental variability within this established cell model.

### 2.3 Subculture and Morphological Observation

When primary cells grown in a 6 cm cell culture dish reached approximately 80% confluency, 1 mL of 0.25% trypsin‐EDTA (Solarbio, T8150) digestion solution was added, followed by a 5 min digestion at room temperature. Subsequently, 5 mL of DMEM/F‐12 medium supplemented with 10% FBS was added to terminate the digestion process. Cells were collected by centrifugation at 1000 rpm, seeded at a density of 10^4^ cells per well in 35 mm culture dishes, and subjected to H&E (haematoxylin and eosin) and Giemsa staining 24 h later. Finally, chromosomal karyotype analysis of the cells was performed. Specifically, when cells in a T25 flask reached confluency, the culture medium was replaced with fresh medium containing colchicine at a final concentration of 0.02 µg/mL, followed by incubation at 37°C for 3–4 h. Cells were then digested, resuspended, and centrifuged at 2600 rpm for 10 min to remove the supernatant. Subsequently, 20 mL of 37°C pre‐warmed hypotonic solution (0.075 mol/L KCl) was added to resuspend the cells, which were then incubated at 37°C for 40 min. Next, 5 mL of 37°C pre‐warmed fixative solution (methanol:glacial acetic acid, 3:1) was added. This was followed by gentle mixing, centrifugation at 2600 rpm for 10 min, and removal of the excess liquid. Another 5 mL of 37°C pre‐warmed fixative solution was added, and the mixture was incubated at room temperature for 15 min before recentrifugation at 2600 rpm for 10 min. Cell suspensions were dropped onto slides from a height exceeding 50 cm, and the slides were baked over an alcohol lamp to ensure cell adhesion. After air‐drying at room temperature, the slides were stained with Giemsa, mounted with neutral resin, and observed under a 1000× oil immersion lens.

### 2.4 Double Immunofluorescence Staining of Cultured Cells

Cells that had been grown on coverslips in 35 mm culture dishes were washed with PBS three times, each time for 3 min, and then fixed with 4% paraformaldehyde for 15 min. Subsequently, the culture dishes were washed with PBS again three times, each time for 3 min. After aspirating the PBS, 5% Bovine Serum Albumin (BSA, Solarbio, A8010) was added dropwise onto the culture dishes, followed by incubation at 37°C for 30 min for blocking. The blocking solution was discarded, and an adequate amount of primary antibody *α*‐SMA (Boster Biological Technology Co., Ltd., dilution at 1:100) was added dropwise to each culture dish, which was then incubated at 37°C for 3 h. The culture dishes were washed with PBS three times, each time for 3 min, and the liquid was aspirated. Then, fluorescent secondary antibody Cy3‐conjugated secondary antibody (ABclonal Technology Co., Ltd., AS015, Wuhan, China; diluted 1:200) was added dropwise and incubated at 37°C for 30 min. The culture dishes were washed with PBS five times, each time for 5 min, and the excess PBS was aspirated. Subsequently, 5% BSA was added dropwise into the culture dishes for blocking at 37°C for another 30 min. The BSA was then aspirated, and an adequate amount of primary antibody CD133 (Boster Biological Technology Co., Ltd. A01767‐3, Wuhan, China) (diluted at 1:100) was added dropwise to each culture dish, which was incubated at 4°C overnight. The culture dishes were warmed to room temperature, washed with PBS three times, each time for 5 min, and the liquid was aspirated. Then, fluorescent secondary antibody Alexa Fluor 488‐conjugated goat anti‐rabbit IgG (IgG/Alexa Fluor 488; Beyotime Biotechnology Co., Ltd., A0423, diluted at 1:200) was added dropwise and incubated at 37°C for 45 min. The culture dishes were washed with PBS three times, each time for 5 min. 4',6‐diamidino‐2‐phenylindole (DAPI, KeyGen Biotech Co., Ltd. KGA301) was added dropwise and incubated in the dark for 5 min, followed by washing with PBS to remove excess DAPI. The culture dishes were sealed with 20% glycerol and observed and photographed under an inverted fluorescence microscope.

### 2.5 Growth Curve Analysis of DPCs

The growth curve of DPCs was determined using a cell counter. Third‐generation DPCs of Hetian sheep in good growth condition were seeded into 6‐well plates at a density of 1×10^4^ cells per well, with 2 mL of culture medium added to each well for cultivation over 7 days. Every 24 h, the cell numbers in three wells were counted using a cell counter to calculate the average value. A cell growth curve was plotted with time as the *x*‐axis and the number of cells per well as the *y*‐axis.

### 2.6 CCK‐8 and EdU Assays for Cell Detection

DPCs were seeded in 96‐well plates at 1×10^3^ cells/well with 10 replicates per group (all replicates are technical, as cells were derived from a single biological donor; *n* = 1). They were treated with 0, 1, 10, 100, or 1000 nM DHT for 48 h. Then, 10 µL of Cell Counting Kit‐8 (CCK‐8, KeyGen Biotech, C0037) reagent was added, cells were incubated at 37°C for 1.5 h, and finally, optical density (OD) at 450 nm was measured to calculate cell viability.

In another setup, DPCs were plated in 6‐well plates at 1×10^4^ cells/well and treated with the same DHT concentrations for 48 h (three replicates each). After adding pre‐warmed 2×5‐Ethynyl‐2'‐deoxyuridine Assay Kit (EdU, Beyotime, C0088) solution and incubating for 24 h, the cells were harvested by trypsinisation. They were fixed, permeabilised, and washed. A click reaction mixture (prepared from Click Additive Solution, CuSO_4_, another relevant component, and Azide 488) was added and incubated at room temperature in the dark for 30 min, then removed by centrifugation. After three washes, the cells were resuspended in PBS and analysed for proliferation using flow cytometry.

### 2.7 Experimental Grouping for DHT Treatment

Cells were treated with different concentrations of Dihydrotestosterone (DHT, Yeyuan Biotechnology Co., Ltd. B34173), 0 and divided into five groups: Group 1 (Control): DPC group (no DHT treatment), Group 2: DPC + 1 nM DHT treatment group (1 nM DHT), Group 3: DPC + 10 nM DHT treatment group (10 nM DHT), Group 4: DPC+100 nM DHT treatment group (100 nM DHT), and Group 5: DPC + 1000 nM DHT treatment group (1000 nM DHT)

Concentration selection rationale: The DHT concentrations (1, 10, 100, and 1000 nM) were selected based on two primary considerations. First, this range encompasses physiologically relevant levels reported in androgen‐responsive tissues and aligns with concentrations widely used in prior in vitro studies investigating androgen effects on dermal papilla cells (e.g., 1–100 nM) (Winiarska et al. 2006; Ho and Ofner 1986; Boivin et al. 2006). Second, extending to 1000 nM served as a supra‐physiological dose to explore potential dose‐dependent effects and saturation responses, a common approach in endocrine‐disrupting compound research (Winiarska et al. 2006; Kang et al. 2015). This concentration gradient (spanning four orders of magnitude) was designed to systematically assess the dose‐response relationship between DHT and DPC proliferation/TGF‐β/Smad signalling.

### 2.8 QPCR‐Based Detection of Gene Expression in DPCs

DPCs were seeded into 60 mm culture dishes at a density of 5×10^5^ cells per dish and allowed to adhere overnight. The cells in each treatment group were then treated with 0 or 1000 nM DHT for 48 h, with three replicate dishes per group. Given that the cells were derived from a single donor, the experimental setup included three technical replicates to control for operational errors. Total RNA was extracted from the cells using TRIzol Reagent (Thermo Fisher Scientific Co., Ltd., 15596‐026), and its concentration and purity were measured to evaluate RNA quality. Following adjustment of the RNA concentration, cDNA was synthesised using the HiScript III RT SuperMix for qPCR (+gDNA wiper) reverse transcription kit (Vazyme Biotech Co., Ltd., R323‐01). The genes, *TGF‐β1, TGF‐β2, TGF‐β3, TGF‐βRII, Smad1, Smad2, Smad3, Smad4, Smad5, Smad6*, and *Smad7*, were detected by quantitative Polymerase Chain Reaction (qPCR, Vazyme Biotech, Q711‐02) using cDNA from cells treated with 0 nM or 1000 nM DHT as templates. The amplification reaction was carried out on a real‐time fluorescence qPCR instrument according to the preset program. Based on the relative quantification method of 2^–ΔΔCt^, with *GAPDH* as the internal reference, the relative mRNA expression levels of the 11 genes were calculated individually. The sequences of all primers used for qPCR amplification are listed in Table 1.

**TABLE 1:** A list showing the sequences of the primers used.

Primer name	Upstream sequence	Downstream sequence	Lengthen (bp)	Genome reference
*TGF‐β1*	GGTGGAATACGGCAACAAA	TGGGCACTGAGGCGAAA	327	XM_042267341.1
*TGF‐β2*	TCCACCATAAAGACAGGAACC	GAGGGCAACAACATTAGCAG	232	XM_012180586.4
*TGF‐βRII*	TGAAGGAAAGAAAGGGGTCT	AGCAGCAAGTCGGGGTTA	118	XM_015097200.3
*TGF‐β3*	CATCAAGAGGAAGCGAGTGG	GGTTGGGCATCCGAAAGA	374	XM_0422
*Smad1*	ATCCCGAGTGGGTGTAGTTT	TCCTGGCGGTGGTATTCT	164	XM_042279199.1
*Smad2*	CCATCTTGCCATTCACTCC	GCTCATCTAATCGTCCTGTTTT	174	XM_042260314.1
*Smad3*	CTACATCGGAGGGGAGGTCT	GGGGTGCTGGTCACAGTTT	288	XM_04217
*Smad4*	ATTTCCTCATGTGATCTATGCC	GTGGATGCTGGATGGTTTG	290	XM_042252512.1
*Smad5*	GATGGATTCACAGACCCTTCG	CCAATATGTCGCCTAGTGTTTTC	104	XM_04226
*Smad6*	CACAGCTCCGGGTGAATT	CCCTGAGGTAGGTCGTAGAAG	159	XM_042274715.1
*Smad7*	CAGCAGTTACCCCATCTTCA	ACGCCTTCTCGTAGTCAAAA	119	XM_042261800.1
*GAPDH*	TGAGATCAAGAAGGTGGTGAA	AAGGTAGAAGAGTGAGTGTCGC	117	XM_042252683.1

### 2.9 Statistical Analysis

Given that the experimental cell model was derived from a single biological individual, all data presented herein correspond to the outcomes of technical replicate measurements performed on this specific cell population. Data were statistically analysed using GraphPad Prism (version 10.1.2 software, San Diego, CA, USA). All data were obtained from at least three independent experiments and are expressed as mean ± standard deviation (SD). For comparison between two groups, an unpaired Student's *t*‐test was used. For comparison among multiple groups, one‐way analysis of variance (ANOVA) was performed, followed by multiple comparisons. Statistical significance was set at *p* < 0.05, and high significance at *p* < 0.01.

## 3 Results

### 3.1 Primary Culture and Morphological Changes of DPCs

**FIGURE 1:**
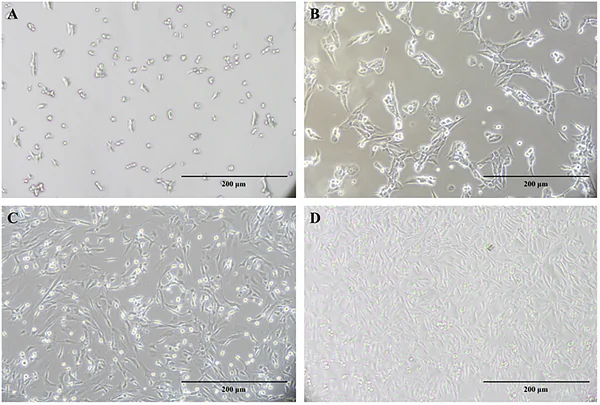
Morphology of DPCs at 200× magnification. (A) On day 3, cells migrated into triangular or short spindle shapes. (B) On day 5, cells gradually enlarged and became long spindle‐shaped. (C) On day 7, cells entered the logarithmic growth phase. (D) On day 12, the growth range no longer expanded, the cell density exceeded 80%, and dead cells began to appear.

DPCs are specialised structures located at the base of hair follicles, often appearing triangular or elliptical under an inverted microscope. During suspension culture, on the 1st day, most DPCs adhered firmly (Figure 1A). By the 3rd day, the cells began to migrate, primarily appearing as fibroblast‐like cells, although some cells also displayed a spindle or triangular shape. On the 5th day, the cells gradually enlarged and adopted a long spindle shape, growing from the edges and radiating outward to the surrounding areas (Figure 1B). By the 7th day, during continued migration, the cells entered the logarithmic growth phase and showed aggregation tendency (Figure 1C). On the 12th day, the cell confluency reached 70–80% (Figure 1D), with a uniform cell morphology. At this stage, the cells were fully fused to form a dense growth area, making it suitable for the first passage.

### 3.2 Cytological and Karyotype Analysis of DPCs

**FIGURE 2:**
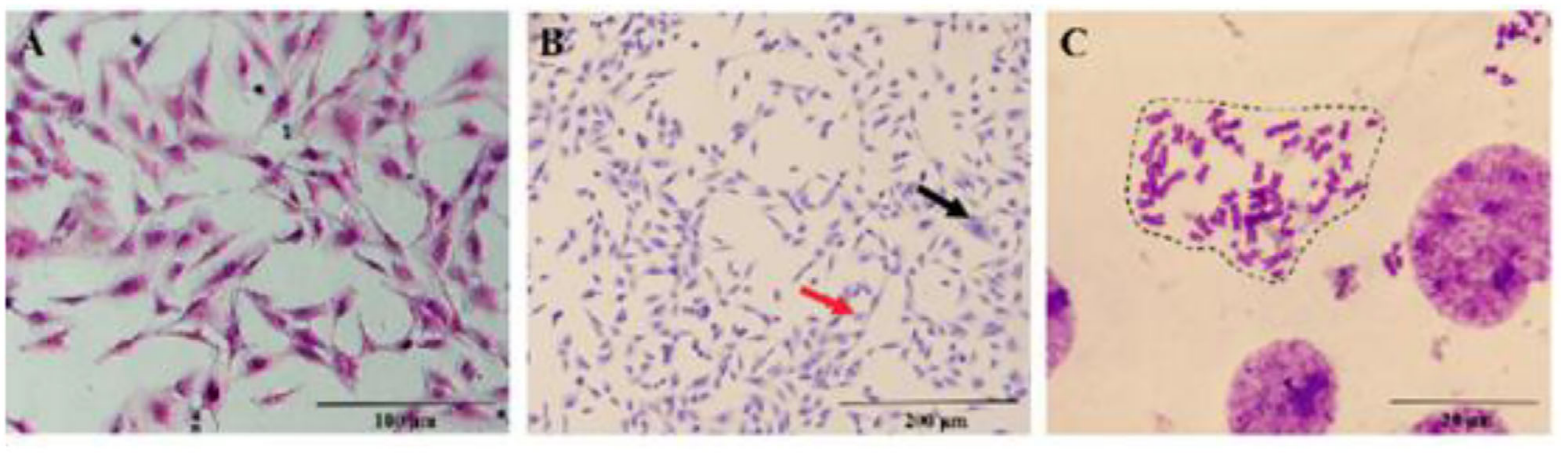
Staining and karyotype analysis of DPCs. (A) H&E staining showed the overall morphology of the cells. (B) Giemsa staining revealed cytoplasmic and nuclear features. The black and red lines indicate the nucleus and cytoplasm, respectively. (C) Chromosome morphology of DPCs from Hetian sheep was observed.

In the third passage, H&E staining revealed that the DPCs displayed an overall purplish‐red colour with abundant nucleoli (Figure 2A). Giemsa staining showed that the cytoplasm was light blue, with one or two nucleoli in the centre of the cell body, which appeared purplish‐red (Figure 2B). Chromosome count analysis of the third‐passage DPCs from Hetian sheep revealed the presence of 54 chromosomes in each cell nucleus (Figure 2C). These included 26 pairs of autosomes and one pair of sex chromosomes. The karyotype of these cells was consistent with that of sheep, confirming that the isolated DPCs were indeed of sheep origin.

### 3.3 Immunofluorescence Identification of DPCs

**FIGURE 3:**
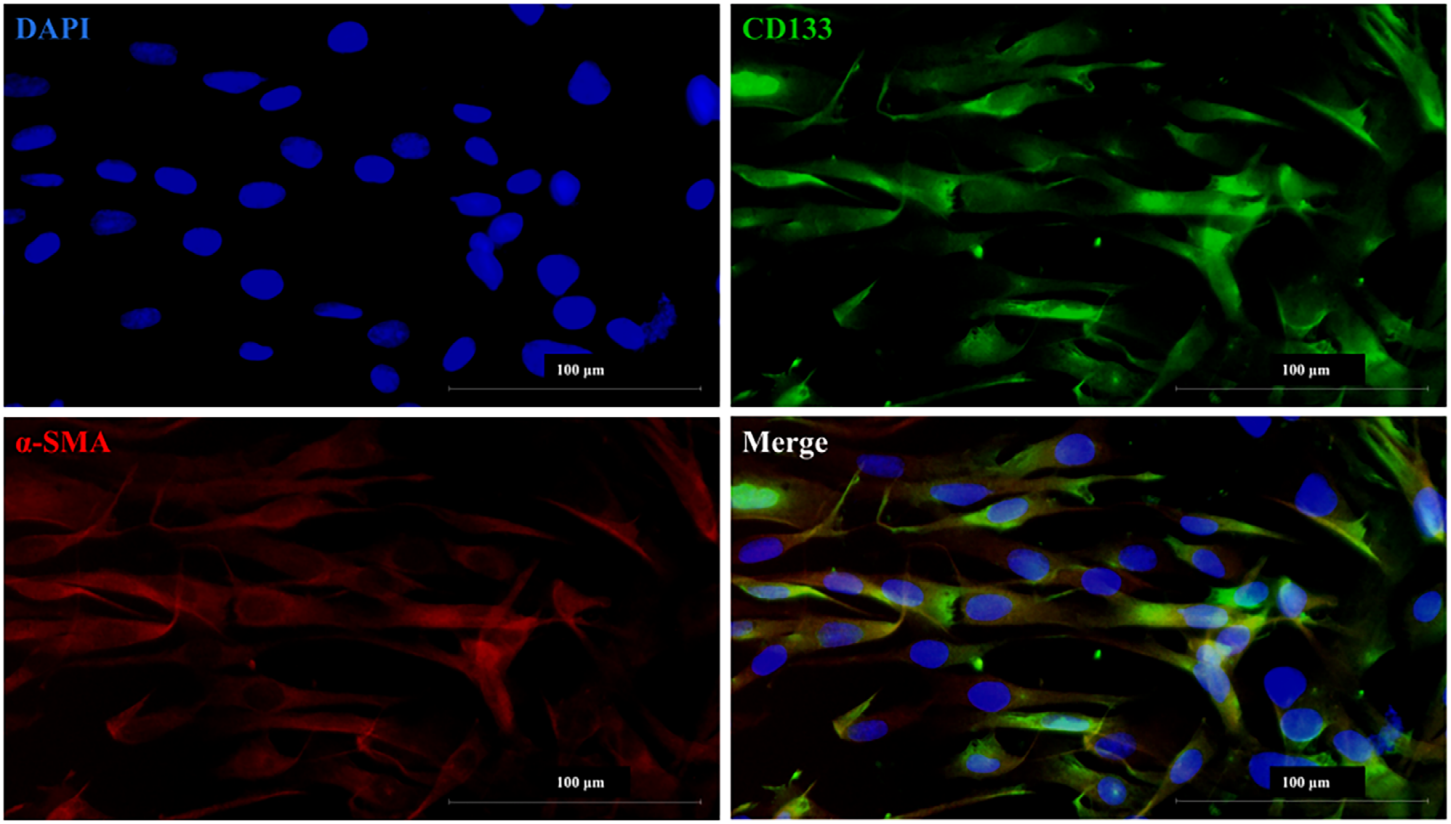
Double immunofluorescence staining of DPCs from Hetian sheep. The different labels and merged images are indicated. Blue fluorescence shows the nuclei, green fluorescence (FITC 488) shows the target protein CD133, and red fluorescence (Cy3) shows the target protein *α*‐SMA.

The immunofluorescence staining results showed that CD133 and *α*‐SMA were positively expressed in the DPCs from Hetian sheep (Figure 3). The merged image demonstrated that the cells were co‐labelled by both antibodies. The co‐expression of the specific markers CD133 and *α*‐SMA confirmed that the cultured cells were DPCs, and their morphology was consistent with that reported in the literature.

### 3.4 Growth Characteristics of Subcultured DPCs

**FIGURE 4:**
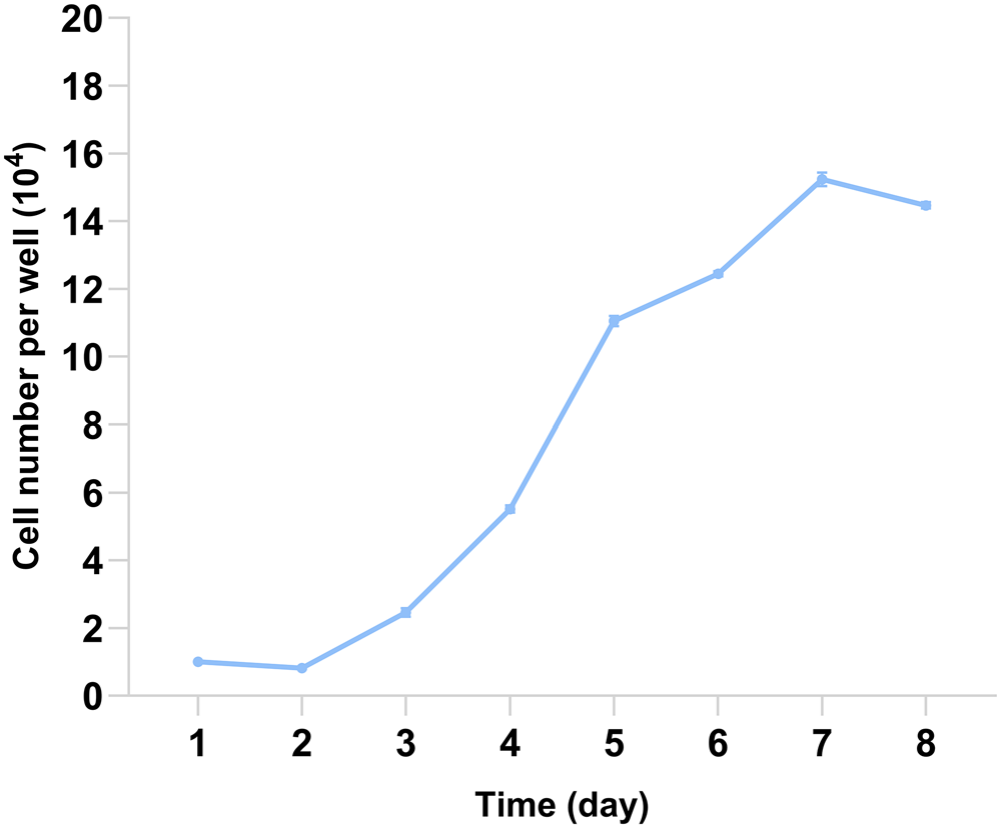
Growth curve of DPCs from Hetian sheep. The curve illustrates the transition through the latent, logarithmic, and plateau phases, reaching peak growth on day 7.

The third passage of subcultured DPCs continued to exhibit robust growth and division, as shown in Figure 4. During the first 1–2 days, the cells were in the latent phase with slow growth. On the 3rd day, the cells began to proliferate rapidly and entered the logarithmic growth phase. Around the 5th–6th day, they reached the plateau phase, and by the 7th day, they reached the peak of growth. Subsequently, the cells gradually aged and died. The results indicated that DPCs from Hetian sheep wool possessed strong growth and division capabilities, making them a suitable source of cells for hair follicle and skin studies. These cells can be used in future studies to investigate the effects of androgens on DPCs.

### 3.5 Effects of DHT Concentration on DPC Proliferation

**FIGURE 5:**
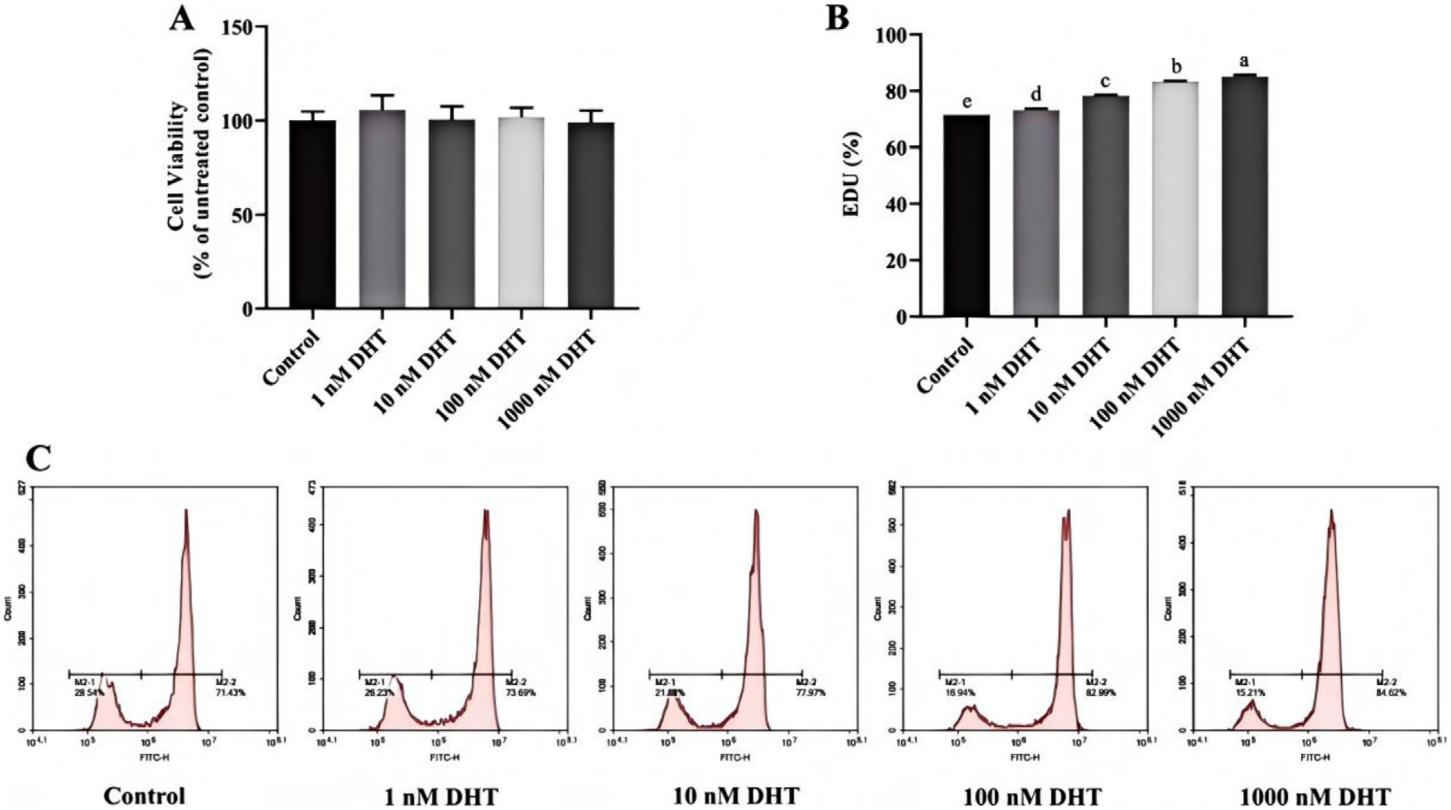
Effects of different DHT concentrations on dermal papilla cell proliferation. (A) Statistical results of cell viability assessed by CCK‐8 (one‐way ANOVA, *p* > 0.05). (B) Statistical results of cell proliferation assessed by EdU. Values with different superscript letters are significantly different (*p* < 0.05), while values with the same superscript letter are not significantly different (*p* > 0.05). (C) Flow cytometry showing DPC proliferation.

The concentrations of DHT used for treatment were selected based on the commonly reported range in previous studies on the effects of androgens on DPCs (Winiarska et al. 2006; Ho and Ofner 1986; Boivin et al. 2006), combined with dose‐exploration pre‐experiments to ensure that the chosen concentrations effectively elicited cellular responses without significant toxicity. DPCs were treated with various concentrations of DHT for 48 h, and their cell viability and proliferation capacity were assessed using CCK‐8 and EdU flow cytometry. As shown in Figure 5, the CCK‐8 results indicated no significant differences in DPC viability or proliferation after 48 h of treatment with different DHT concentrations (one‐way ANOVA, *p* > 0.05). However, in the EdU flow cytometry analysis, it was noted that as the DHT concentration increased, the cell proliferation capacity gradually improved, reaching a peak at 1000 nM DHT. Statistical analysis using one‐way ANOVA revealed a significant overall effect of DHT concentration on EdU incorporation (*p* < 0.05), with post hoc comparisons indicating a significant difference between the 1000 nM group and the control group (*p* < 0.05).

### 3.6 Effect of DHT Treatment on Gene Expression in DPCs

**FIGURE 6:**
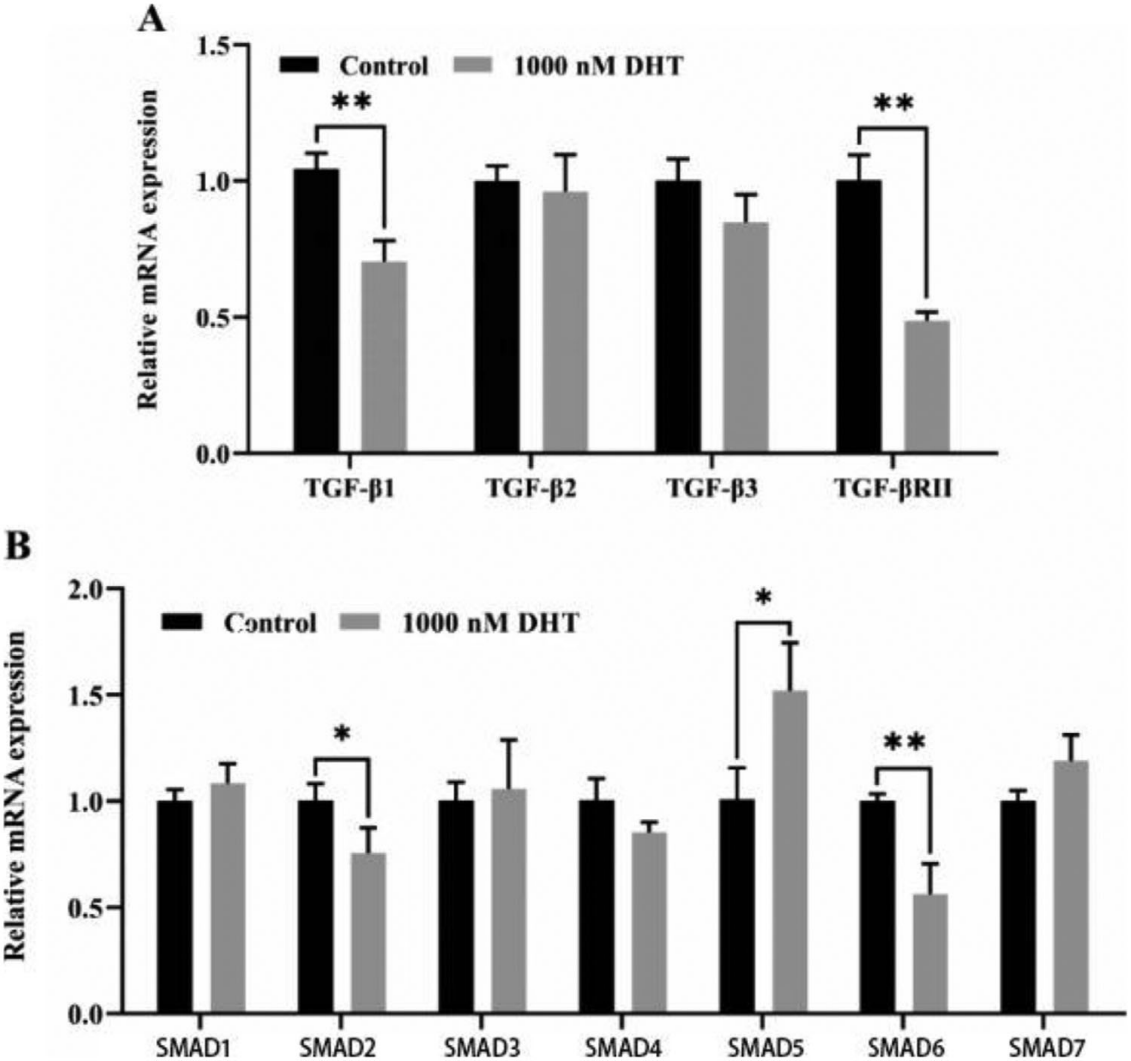
Effect of DHT treatment on gene expression levels in DPCs. (A) Relative mRNA expression of *TGF‑β* superfamily ligand and receptor genes. (B) Relative mRNA expression of *SMAD* family genes. Data are presented as the mean ± SD, *p < 0.05; **p < 0.01.

Cell proliferation results indicated that when 1000 nM DHT was added during cell culture, the cell proliferation capacity was strongest. Therefore, DPCs treated with 1000 nM DHT and untreated DPCs were cultured separately for 48 h to analyse the effects of DHT treatment on the expression of four genes from the TGF family and seven genes from the SMAD family. As shown in Figure 6A, compared to the control group, the expression of TGF‐β1 and TGF‐*β*RII in the 1000 nM DHT group was significantly downregulated, with extremely significant differences (Student's *t*‐test, *p < 0.01). There were no significant differences in the expression of *TGF*‐β*2* and *TGF‐*β*3* between the 1000 nM DHT group and the control group. As shown in Figure 6B, within the SMAD gene family, 1000 nM DHT treatment reduced the expression of Smad2 and Smad6, increased the expression of *Smad5*, and had no significant effect on the expression of *Smad1*, *Smad3*, *Smad4*, and *Smad7* (Student's *t*‐test, *p* values as indicated).

## 4 Discussion

DPCs act as key regulatory elements in the hair follicle microenvironment. They function by secreting various signalling molecules, such as growth factors and cytokines, that act on hair follicle epithelial cells (Liu et al. 2020). These molecules regulate epithelial cell proliferation and differentiation, as well as hair follicle regeneration and maintenance. DPCs function as the regulatory centre of the entire hair follicle and play a crucial role in hair follicle development (Luo et al. 2023). DPCs are essential not only for inducing and maintaining the hair follicle growth cycle but also for determining hair follicle size and the duration of the anagen phase. Due to the high abundance of DPCs in vivo and the technical difficulty of microdissection, researchers have focused on isolating and culturing DPCs in vitro with hair‐inducing potential (Vinayak et al. 2025). Many studies have reported in vitro DPC culture from human scalp hair follicles, *murine vibrissae* follicles, and follicles from macaques, minks, goats, and rex rabbits (Elkiki et al. 2021). Immortalised dermal papilla cell lines have also been established in humans. Culturing DPCs from Hetian sheep provides an in vitro cellular model for screening factors that regulate hair follicle development and wool growth. It also supports the study of mechanisms by which androgens regulate hair follicle development and growth (Luo et al. 2023).

In the in vitro culture of DPCs, a microdissection method was used to isolate the dermal papilla (DP) from primary lamb follicles. This was combined with enzymatic digestion to disaggregate the DP into a cell suspension. The suspension was then filtered through a cell sieve, which significantly shortened the migration time of newly formed DPCs and improved separation efficiency and culture success rates (Wang et al. 2023). In the culture of Hetian sheep DPCs, the cells adhered rapidly to the substrate and maintained strong proliferative activity. Their morphology was spindle‐shaped and arranged in a multipolar pattern while retaining the characteristic of aggregative growth. This aggregative growth can induce hair growth in vivo, but it is often lost after multiple passages (Liu et al. 2020). Therefore, the early‐passage cells used in this study retained the ability to induce hair growth and better reflected DPC behaviour in the in vivo skin environment. Typically, isolated and cultured DPCs require immunofluorescence identification using markers such as AP, *α*‐SMA, Versican, Corin, and CD133. Prior studies have confirmed that *α*‐SMA and CD133 show positive immunoreactivity in the DPCs of cashmere goats. *α*‐SMA also exhibits positive staining in DPCs from rex rabbits (Jia et al. 2023; Zhang et al. 2024; Huacachino et al. 2024). Zhao et al. demonstrated through skin reconstitution assays that CD133^+^ DPCs are the primary cellular driver of hair follicle neogenesis (Zhao et al. 2025). In this study, positive labelling with CD133 antibodies indicated that the cultured cells represented this pivotal hair‐inductive subpopulation. Furthermore, positive immunolabelling for *α*‐smooth muscle actin (*α*‐SMA), a conserved marker that confers contractile properties and signifies myofibroblast‐like traits in DPCs, was confirmed (Jia et al. 2023). Together, the expression of these two definitive markers provides compelling evidence for the successful isolation of authentic DPCs with typical characteristics. This study demonstrates that Hetian sheep DPCs can be labelled by both *α*‐SMA and CD133 antibodies, confirming that the cultured cells were indeed DPCs from Hetian sheep.

This study examined the effects of different concentrations of DHT on DPC proliferation. It was found that DHT promoted cell proliferation, with higher concentrations resulting in greater proliferation, while significantly inhibiting *TGF*‐*β* expression. TGF‐β promotes collagen secretion and enhances both the maintenance and hair‐inducing capacity of DPCs (Yamane et al. 2022; Kang et al. 2015). Androgens play a crucial role in maintaining the epidermal and hair growth cycle. They also regulate the proliferation and differentiation of various hair follicle cells by modulating the TGF‐β signalling pathway. In the TGF‐β/Smad signalling system, the expression levels of membrane receptor protein kinase families and receptor substrate families (Smad proteins) are key regulators of hair follicle development and hair growth (Fabregat et al. 2016). Androgens may affect hair follicle development by modulating the expression of specific signalling components within the Smad protein family. Previous research has shown that DHT delays the cell cycle progression of immortalised human scalp DPCs without impacting proliferation (Humphreys et al. 2020; Elkiki et al. 2021; Wang et al. 2023; Yamane et al. 2022). Due to differences across body regions, androgens may have varying effects on hair follicles in human scalp skin compared to other areas. For example, androgenetic alopecia is limited to genetically sensitive regions of the human scalp (Yamane et al. 2022) and does not occur in normal skin. Therefore, the response of human scalp DPCs to androgens may differ from that of Hetian sheep DPCs. Analysis of DHT‐treated DPC gene expression demonstrated that androgens inhibited the expression of the *TGF‐β1* gene. Studies have confirmed that androgens reduce *TGF‐β1* expression in DPCs and suppress the proliferation of co‐cultured keratinocytes (Ying et al. 2024; Gou et al. 2025), resulting in a 50% reduction in the keratinocyte proliferation rate. These findings suggest that androgens may suppress hair follicle growth and development by downregulating *TGF‐β1* expression in DPCs. Androgen stimulation increased *Smad5* expression and decreased *Smad6* expression. *Smad5* mediates BMP signalling, while *Smad6* inhibits it. Thus, androgens may indirectly influence BMP signalling by regulating *Smad5* and *Smad6* levels. BMP signalling regulates the hair follicle cycle, delays follicle development, and can alter follicle size and spacing. In summary, our results suggest that androgens may regulate hair follicle growth and development by inhibiting the *TGF*‐β/*Smad* signalling pathway.

While the present study provides novel insights into the role of DHT in promoting DPC proliferation via the TGF‐β/Smad pathway, it is important to acknowledge certain limitations. First, we have not yet performed additional validation experiments using complementary marker genes at the protein level (e.g., Western blot) or other proliferation‐associated markers beyond CCK‐8 and EdU. Such experiments could further corroborate the observed proliferative effects of DHT on Hetian sheep DPCs. Second, our conclusions regarding the inhibitory effect of DHT on the TGF‐β/Smad pathway are based on gene expression data alone. Rescue experiments – such as exogenous TGF‐β1 supplementation or pharmacological inhibition of Smad signalling in the presence of DHT – would provide stronger functional evidence for the causal role of this pathway in mediating DHT effects. Future studies should incorporate these approaches to strengthen the mechanistic link between DHT and TGF‐β/Smad signalling. Additionally, investigating the in vivo relevance of these findings through animal models or 3D culture systems could further elucidate the physiological implications for wool growth and hair follicle biology.

## 5 Conclusion

We isolated DPCs from Hetian sheep and established a reliable culture system. It was observed that androgen promotes the proliferation of DPCs by inhibiting the expression of *TGF*‐β/*Smad* pathway genes, thereby influencing hair follicle development. An in vitro model for sheep‐based research was established, linking androgen regulation with hair follicle development. These findings provide a theoretical basis for increasing wool yield in sheep and offer new research directions for studying androgenetic alopecia.

## Author Contributions

Qinqin Wang: software, writing – original draft preparation. Xuan Liu: translation, visualisation, writing – review and editing. Ruijun Shi and Shuwei Li: methodology, validation, resources, supervision, project administration, and funding acquisition. Zhiya Feng: data curation. Jianping Zhang: visualisation. Chunyang Li: data proofreading. All the authors have read and agreed to the published version of the manuscript.

## Funding

This study was funded by the Guide Science and Technology Plan Project of the Production and Construction Corps (NO.2023ZD115); Young Doctor Project of the ‘Tianchi Talents’ Introduction Plan (NO.524307007); Cultivation Project of ‘Tianshan Talents’ – Leading Talents in Scientific and Technological Innovation (NO.2023TSYCLJ0057).

## Ethics Statement

All the procedures for animal testing were performed in accordance with the Chinese Guide for the Care and Use of Animals for Research (GB14925‐2001). All experimental protocols were approved by the Laboratory Animal Center of Tarim University and the Ethics Committee of Science and Technology of Tarim University (approval number: PB20260112003).

## Consent

All co‐authors consented for publication.

## Conflicts of Interest

The authors declare no conflicts of interest.

## Data Availability

The raw research datasets generated and analysed in this study are not publicly available due to laboratory data management restrictions.
